# Supplementary material for: The serum vitamin D levels alleviate the influence of dietary inflammation on frailty: A cross-sectional analysis in the U.S. older adults
Source: PLoS One. 2025 Jun 30;20(6):e0327251. doi: 10.1371/journal.pone.0327251 (PMC12208477; doi:10.1371/journal.pone.0327251)
Supplement: S1 File — S1 Table Variables in the 49-Item Frailty Index and Their Respective Scorings. S2 Table The relationship between DII and frailty was observed by subgroup analysis after weighting. S1 Data. (ZIP) [file pone.0327251.s001.zip › Supplementary materials/S1 Table.docx]

**Supplementary Table 1**

S1 Table Variables in the 49-Item Frailty Index and Their Respective Scorings.

| **Variable** | **Scoring** |  |
| --- | --- | --- |
| Cognition |  |  |
| 1. Experience confusion/memory problems | Yes=1, No = 0 |  |
| Dependence |  |  |
| 2. Managing money | Difficulty =1, No Difficulty = 0 |  |
| 3. Stooping, crouching, kneeling | Difficulty =1, No Difficulty = 0 |  |
| 4. Lifting or carrying | Difficulty =1, No Difficulty = 0 |  |
| 5. House chore | Difficulty =1, No Difficulty = 0 |  |
| 6. Preparing meals | Difficulty =1, No Difficulty = 0 |  |
| 7. Standing up from armless chair | Difficulty =1, No Difficulty = 0 |  |
| 8. Getting in and out of bed difficulty | Difficulty =1, No Difficulty = 0 |  |
| 9. Using fork, knife, drinking from cup | Difficulty =1, No Difficulty = 0 |  |
| 10. Dressing yourself | Difficulty =1, No Difficulty = 0 |  |
| 11. Standing for long periods difficulty | Difficulty =1, No Difficulty = 0 |  |
| 12. Grasp/holding small objects | Difficulty =1, No Difficulty = 0 |  |
| 13. Attending social event | Difficulty =1, No Difficulty = 0 |  |
| 14. Push or pull large objects | Difficulty =1, No Difficulty = 0 |  |
| 15. Walking for a quarter mile difficulty | Difficulty =1, No Difficulty = 0 |  |
| 16. Walking up 10 steps difficulty | Difficulty =1, No Difficulty = 0 |  |
| Depressive Symptoms |  |  |
| 17. Have little interest in doing things | Nearly every day = 1, More than half the days =0.66, Several days =0.33, Not at all =0 |  |
| 18. Feeling down, depressed, or hopeless | Nearly every day = 1, More than half the days =0.66, Several days =0.33, Not at all =0 |  |
| 19. Trouble sleeping or sleeping too much | Nearly every day = 1, More than half the days =0.66, Several days =0.33, Not at all =0 |  |
| 20. Feeling tired or having little energy | Nearly every day = 1, More than half the days =0.66, Several days =0.33, Not at all =0 |  |
| 21. Poor appetite or overeating | Nearly every day = 1, More than half the days =0.66, Several days =0.33, Not at all =0 |  |
| 22. Feeling bad about yourself | Nearly every day = 1, More than half the days =0.66, Several days =0.33, Not at all =0 |  |
| 23. Trouble concentrating on things | Nearly every day = 1, More than half the days =0.66, Several days =0.33, Not at all =0 |  |
| Comorbidities |  |  |
| 24. Arthritis | Yes = 1, Suspect=0.5, No=0 |  |
| 25. Thyroid problems | Yes = 1, Suspect=0.5, No=0 |  |
| 26. Chronic bronchitis | Yes = 1, Suspect=0.5, No=0 |  |
| 27. Cancer | Yes = 1, Suspect=0.5, No=0 |  |
| 28. Congestive heart failure | Yes = 1, Suspect=0.5, No=0 |  |
| 29. Coronary heart disease | Yes = 1, Suspect=0.5, No=0 |  |
| 30. Angina | Yes = 1, Suspect=0.5, No=0 |  |
| 31. Heart attack | Yes = 1, Suspect=0.5, No=0 |  |
| 32. Stroke | Yes = 1, Suspect=0.5, No=0 |  |
| 33. Blood pressure | Yes = 1, Suspect=0.5, No=0 |  |
| 34. Diabetes | Yes = 1, Suspect=0.5, No=0 |  |
| 35. weak/failing kidneys | Yes = 1, Suspect=0.5, No=0 |  |
| 36. Urinary Leakage | Yes = 1, Suspect=0.5, No=0 |  |
| Hospital Utilization and Access to Care |  |  |
| 37. Self-rated health | Fair, poor = 1, Excellent, Very good, good = 0 |  |
| 38. Health now compared with 1 year ago | Worse= 1, About the same, better =0 |  |
| 39. Overnight hospital patient in past year | Yes= 1,No=0 |  |
| 40. Frequency of health care use during past year | None =0, 1-5 =0,5, More than 5 =1 |  |
| 41. Number of prescribed medications Physical Performance and Anthropometry | None=0,1-4 =0.5, 5 and more =1 |  |
| 42. Body mass index | <18.5, ≥30=1   1. <30=0.5 2. 18.5-25 =0 |  |
| 43. Handgrip strength | MALE:  For BMI≤24, GS≤29  For BMI≤24.1-28, GS≤30  For BMI>28, GS≤32=1 | FEMALE:  For BMI≤23, GS≤17  For BMI≤23.1-26, GS≤17.3  For BMI26.1-29, GS≤18  For BMI>29, GS≤21=1 |
| Laboratory Values |  |  |
| 44. Glycohemoglobin (%) | 0%-5.7%=0, >5.7%=1 |  |
| 45. Red blood cell count (million cells/mL) | M:4.7-6.1=0, Other=1 | F:4.2-5.4=0, Other=1 |
| 46. Hemoglobin (g/dL) | M:13.5-18=0, Other=1 | F:12-16=0, Other=1 |
| 47. Red cell distribution width (%) | 11.6-14.6=0, Other=1 |  |
| 48. Lymphocyte percent (%) | 20-40=0，Other=1 |  |
| 49. Segmented neutrophils percent (%) | 40-80=0，Other=1 |  |
