# Supplementary material for: The serum vitamin D levels alleviate the influence of dietary inflammation on frailty: A cross-sectional analysis in the U.S. older adults
Source: PLoS One. 2025 Jun 30;20(6):e0327251. doi: 10.1371/journal.pone.0327251 (PMC12208477; doi:10.1371/journal.pone.0327251)
Supplement: S1 File — S1 Table Variables in the 49-Item Frailty Index and Their Respective Scorings. S2 Table The relationship between DII and frailty was observed by subgroup analysis after weighting. S1 Data. (ZIP) [file pone.0327251.s001.zip › Supplementary materials/S2 Table.docx]

**Supplementary Table 1**

S2 Table The relationship between DII and frailty was observed by subgroup analysis after weighting.

| **Subgroup** | **Model 1** | | |  | **Model 2** | | |  | **Model 3** | | |
| --- | --- | --- | --- | --- | --- | --- | --- | --- | --- | --- | --- |
|  | **OR** | **95%CI** | ***P*** |  | **OR** | **95%CI** | ***P*** |  | **OR** | **95%CI** | ***P*** |
| **Sex** |  |  |  |  |  |  |  |  |  |  |  |
| Male | 1.21 | (1.10~1.32) | <0.001 |  | 1.18 | (1.07~1.31) | 0.001 |  | 1.16 | (1.04~1.29) | 0.007 |
| Female | 1.12 | (1.02~1.22) | 0.012 |  | 1.05 | (0.96~1.16) | 0.232 |  | 1.04 | (0.93~1.18) | 0.404 |
| **Age** |  |  |  |  |  |  |  |  |  |  |  |
| [60~70) | 1.23 | (1.12~1.35) | <0.001 |  | 1.17 | (1.04~1.32) | 0.008 |  | 1.15 | (1.01~1.31) | 0.029 |
| [70~80] | 1.11 | (1.03~1.93) | 0.005 |  | 1.07 | (0.99~1.16) | 0.076 |  | 1.08 | (0.97~1.19) | 0.125 |
