# Supplementary material for: The serum vitamin D levels alleviate the influence of dietary inflammation on frailty: A cross-sectional analysis in the U.S. older adults
Source: PLoS One. 2025 Jun 30;20(6):e0327251. doi: 10.1371/journal.pone.0327251 (PMC12208477; doi:10.1371/journal.pone.0327251)
Supplement: Attachment — Submitted filename: Response to Reviewers.docx [file pone.0327251.s002.docx]

**Dear Editors and reviewers,**

**We are grateful for the valuable comments and suggestions given by the reviewers and editors, and found them very useful in improving our manuscript, the submission ID is PONE-D-24-52566.**

**We give our responses to the reviewers in this letter. We hope that after our replies, our manuscript will be considered by PLOS ONE.**

**Q1:** Please ensure that your manuscript meets PLOS ONE' s style requirements, including those for file naming.

**Response:** Thank you for your careful review. We have checked the manuscript item by item to ensure full compliance with the PLOS ONE format specification.

**Q2:** We note that the grant information you provided in the ‘Funding Information’ and ‘Financial Disclosure’ sections do not match.

**Response:** Thank you for your careful review. Due to an error during the online submission process, the funding information was incorrectly stated as "The authors received no specific funding for this work." As a result, the 'Funding Information' and 'Financial Disclosure' sections do not match. This work was supported by Scientific Research Funding Project of Ningxia Medical University (grant number XT2022014). We kindly request your assistance in updating the online submission form to reflect the correct funding information provided above.

**Q3:** Thank you for stating the following financial disclosure:

“The author(s) received no specific funding for this work.”

At this time, please address the following queries:

a) Please clarify the sources of funding (financial or material support) for your study. List the grants or organizations that supported your study, including funding received from your institution.

b) State what role the funders took in the study. If the funders had no role in your study, please state: “The funders had no role in study design, data collection and analysis, decision to publish, or preparation of the manuscript.”

c) If any authors received a salary from any of your funders, please state which authors and which funders.

d) If you did not receive any funding for this study, please state: “The authors received no specific funding for this work.” Please include your amended statements within your cover letter; we will change the online submission form on your behalf.

**Response:** Thank you for your feedback. We would like to clarify the funding information for our study: This work was supported by Scientific Research Funding Project of Ningxia Medical University (grant number XT2022014). Due to an error during the online submission process, the funding information was incorrectly stated as "The authors received no specific funding for this work." We kindly request your assistance in updating the online submission form to reflect the correct funding information provided above.

**Q4:** In this instance it seems there may be acceptable restrictions in place that prevent the public sharing of your minimal data. However, in line with our goal of ensuring long-term data availability to all interested researchers, PLOS’ Data Policy states that authors cannot be the sole named individuals responsible for ensuring data access (http://journals.plos.org/plosone/s/data-availability#loc-acceptable-data-sharing-methods).

Data requests to a non-author institutional point of contact, such as a data access or ethics committee, helps guarantee long term stability and availability of data. Providing interested researchers with a durable point of contact ensures data will be accessible even if an author changes email addresses, institutions, or becomes unavailable to answer requests.

Before we proceed with your manuscript, please also provide non-author contact information (phone/email/hyperlink) for a data access committee, ethics committee, or other institutional body to which data requests may be sent. If no institutional body is available to respond to requests for your minimal data, please consider if there any institutional representatives who did not collaborate in the study, and are not listed as authors on the manuscript, who would be able to hold the data and respond to external requests for data access? If so, please provide their contact information (i.e., email address). Please also provide details on how you will ensure persistent or long-term data storage and availability.

**Response:** Thank you for your valuable comments and suggestions. We have updated the Data Availability Statement in the article to make it clear that the study data is provided as supplementary materials (Data S1), and we have updated the Ethics Statement section to ensure that all research activities are ethically compliant and that appropriate ethical approvals have been obtained.

**Q5:** PLOS requires an ORCID iD for the corresponding author in Editorial Manager on papers submitted after December 6th, 2016. Please ensure that you have an ORCID iD and that it is validated in Editorial Manager. To do this, go to ‘Update my Information’ (in the upper left-hand corner of the main menu), and click on the Fetch/Validate link next to the ORCID field. This will take you to the ORCID site and allow you to create a new iD or authenticate a pre-existing iD in Editorial Manager.

**Response:** Thank you for your comment regarding the ORCID iD requirement. Corresponding author have already obtained an ORCID iD and validated it in Editorial Manager. The ORCID iD is [0000-0002-4316-3489], and it has been successfully authenticated in the system.

Reviewer #1

**Q1:** The introduction seems to be long and could be further shortened so that readers can grasp the core content of the study more quickly. For example, the third and fourth paragraphs could be integrated into one paragraph.

**Response:** Thank you very much for your professional advice. The introduction section has been streamlined to allow readers to access the core findings of this study more promptly and efficiently. We have made revisions and marked in red in the introduction part.

**Q2:** The Discussion section should refer to the latest literature in the past 3-5 years to explore in more detail how serum vitamin D levels mediate the relationship between DII and frailty and how these findings relate to existing theories of immune and inflammatory responses.

**Response:** We have updated the relevant literature, referencing the latest studies from the past 3-5 years. Additionally, we have provided a more detailed exploration of how serum vitamin D levels mediate the relationship between the DII and frailty, as well as how these findings relate to existing theories of immune and inflammatory responses. We have also elaborated on the potential mechanisms involved. All revisions were marked in red in the discussion part, can be seen in the lines 298-317, pages 14-15.

**Q3:** In lines 154-155, "Ratio of family income to poverty (RIP)" should be changed to "Ratio of family income to poverty (PIR)". Please see the full text.

**Response:** Many thanks. We have revised the “Ratio of family income to poverty (PIR)” as“Ratio of family income to poverty (RIP)”.

**Q4:** Please add a flow chart of the study participants to this article.

**Response:** Thank you for your valuable suggestion to improve the clarity of participant flow. We have added the participant flow chart (Figure 1) in the Study Design and Participants section, which systematically presents the complete process of sample selection and final inclusion in the analysis.

**Q5:** What is the basis for the adjustment of confounders included in this article? In addition, it is recommended that baseline data such as BMI, diabetes, and hypertension should be included.

**Response:** Thank you very much for your professional advice. I have added the variables of BMI, height, weight, energy intake, diabetes and hypertension in the baseline Characteristics Table. For details, see Table 1 (Participant Characteristics). Secondly, in this study, we selected several covariates associated with dietary inflammation and frailty based on previously published research to adjust for potential confounders. The literature is as follows:

[1] Ma R, Zhou X, Zhang G, Wu H, Lu Y, Liu F, Chang Y, Ding Y. Association between composite dietary antioxidant index and coronary heart disease among US adults: a cross-sectional analysis. BMC Public Health. 2023 Dec 5;23(1):2426. doi: 10.1186/s12889-023-17373-1. PMID: 38053099; PMCID: PMC10699074.
[2] Sun M, Wang L, Wang X, Tong L, Fang J, Wang Y, Yang Y, Li B. Interaction between sleep quality and dietary inflammation on frailty: NHANES 2005-2008. Food Funct. 2023 Jan 23;14(2):1003-1010. doi: 10.1039/d2fo01832b. PMID: 36546877.
[3] Mao Y, Weng J, Xie Q, Wu L, Xuan Y, Zhang J, Han J. Association between dietary inflammatory index and Stroke in the US population: evidence from NHANES 1999-2018. BMC Public Health. 2024 Jan 2;24(1):50. doi: 10.1186/s12889-023-17556-w. PMID: 38166986; PMCID: PMC10763382.

**Q6:** In the future, further prospective studies are needed to determine the exact relationship between DII and frailty.

**Response:** We fully agree with your point regarding the need for further prospective studies to determine the exact relationship between DII and frailty. This is indeed a crucial area for future research.

We have incorporated your suggestion into the limitation part, and highlighted in red, and the revised text is transcribed below:

“The study acknowledges the presence of several strengths and limitations. Broadening the knowledge regarding the underlying mechanisms in the relationship between DII and frailty of older adults, particularly the mediating role of serum vitamin D in the relationship between DII and frailty was explored. However, several limitations of the study must also be clarified. Firstly, given the type of study in which cross-sectional studies are conducted, causation cannot be determined. In the future, further prospective studies are needed to determine the exact relationship between DII and frailty. Secondly…

Reviewer #2:

**Q1:** In several instances throughout the manuscript, the authors use the term effect, such as in the objective stated in the abstract: "The current study aimed to explore the mediating effect of vitamin D levels in the link between DI and frailty." However, since this is a cross-sectional study, causality cannot be determined. Therefore, it would be more appropriate to replace effect with relationship or association to better reflect the study design and its limitations.

**Response:** Thank you very much for your insightful comments. We have carefully revised the manuscript to replace “effect” with “association” throughout. And highlighted in red in the manuscript.

**Q2:** I suggest that the authors provide a more detailed explanation of the parameters used for frailty classification. Clarifying the specific criteria or thresholds applied in the frailty index would improve the transparency and reproducibility of the study.

**Response:** Yes, to enhance the transparency and reproducibility of our frailty classification methodology, we have now added a detailed description of the parameters, criteria, and thresholds used in the frailty index. Please refer to the supplementary materials for further details. We have revised this part, highlighted in red in the manuscript, and the revised text is transcribed below:

“The frailty index comprised 49 deficits spanning multiple systems [45], including Cognition (Experience confusion/memory problems), Dependence(Managing money, Stooping, crouching, kneeling, and Lifting or carrying; etc., 15 items), Depressive Symptoms (Have little interest in doing things, Trouble sleeping or sleeping too much and Feeling tired or having little energy; etc., 7 items), Comorbidities (Arthritis, Thyroid problems and Chronic bronchitis; etc., 13 items), Hospital Utilization and Access to Care (Self-rated health, Health now compared with 1 year ago and Overnight hospital patient in past year; etc., 6 items), Laboratory Values (Glycohemoglobin, Red blood cell count and Hemoglobin; etc., 6 items) (Table S1).” In this study, fit and vulnerable (i.e., FI≤0.2) were combined into the no-frailty group, while mildly frail and moderately/severely frail (i.e., FI > 0.2) were combined into the frailty group.

**Supplementary Table 1**

Variables in the 49-Item Frailty Index and Their Respective Scorings.

| **Variable** | **Scoring** |  |
| --- | --- | --- |
| Cognition |  |  |
| 1. Experience confusion/memory problems | Yes=1, No = 0 |  |
| Dependence |  |  |
| 2. Managing money | Difficulty =1, No Difficulty = 0 |  |
| 3. Stooping, crouching, kneeling | Difficulty =1, No Difficulty = 0 |  |
| 4. Lifting or carrying | Difficulty =1, No Difficulty = 0 |  |
| 5. House chore | Difficulty =1, No Difficulty = 0 |  |
| 6. Preparing meals | Difficulty =1, No Difficulty = 0 |  |
| 7. Standing up from armless chair | Difficulty =1, No Difficulty = 0 |  |
| 8. Getting in and out of bed difficulty | Difficulty =1, No Difficulty = 0 |  |
| 9. Using fork, knife, drinking from cup | Difficulty =1, No Difficulty = 0 |  |
| 10. Dressing yourself | Difficulty =1, No Difficulty = 0 |  |
| 11. Standing for long periods difficulty | Difficulty =1, No Difficulty = 0 |  |
| 12. Grasp/holding small objects | Difficulty =1, No Difficulty = 0 |  |
| 13. Attending social event | Difficulty =1, No Difficulty = 0 |  |
| 14. Push or pull large objects | Difficulty =1, No Difficulty = 0 |  |
| 15. Walking for a quarter mile difficulty | Difficulty =1, No Difficulty = 0 |  |
| 16. Walking up 10 steps difficulty | Difficulty =1, No Difficulty = 0 |  |
| Depressive Symptoms |  |  |
| 17. Have little interest in doing things | Nearly every day = 1, More than half the days =0.66, Several days =0.33, Not at all =0 |  |
| 18. Feeling down, depressed, or hopeless | Nearly every day = 1, More than half the days =0.66, Several days =0.33, Not at all =0 |  |
| 19. Trouble sleeping or sleeping too much | Nearly every day = 1, More than half the days =0.66, Several days =0.33, Not at all =0 |  |
| 20. Feeling tired or having little energy | Nearly every day = 1, More than half the days =0.66, Several days =0.33, Not at all =0 |  |
| 21. Poor appetite or overeating | Nearly every day = 1, More than half the days =0.66, Several days =0.33, Not at all =0 |  |
| 22. Feeling bad about yourself | Nearly every day = 1, More than half the days =0.66, Several days =0.33, Not at all =0 |  |
| 23. Trouble concentrating on things | Nearly every day = 1, More than half the days =0.66, Several days =0.33, Not at all =0 |  |
| Comorbidities |  |  |
| 24. Arthritis | Yes = 1, Suspect=0.5, No=0 |  |
| 25. Thyroid problems | Yes = 1, Suspect=0.5, No=0 |  |
| 26. Chronic bronchitis | Yes = 1, Suspect=0.5, No=0 |  |
| 27. Cancer | Yes = 1, Suspect=0.5, No=0 |  |
| 28. Congestive heart failure | Yes = 1, Suspect=0.5, No=0 |  |
| 29. Coronary heart disease | Yes = 1, Suspect=0.5, No=0 |  |
| 30. Angina | Yes = 1, Suspect=0.5, No=0 |  |
| 31. Heart attack | Yes = 1, Suspect=0.5, No=0 |  |
| 32. Stroke | Yes = 1, Suspect=0.5, No=0 |  |
| 33. Blood pressure | Yes = 1, Suspect=0.5, No=0 |  |
| 34. Diabetes | Yes = 1, Suspect=0.5, No=0 |  |
| 35. weak/failing kidneys | Yes = 1, Suspect=0.5, No=0 |  |
| 36. Urinary Leakage | Yes = 1, Suspect=0.5, No=0 |  |
| Hospital Utilization and Access to Care |  |  |
| 37. Self-rated health | Fair, poor = 1, Excellent, Very good, good = 0 |  |
| 38. Health now compared with 1 year ago | Worse= 1, About the same, better =0 |  |
| 39. Overnight hospital patient in past year | Yes= 1,No=0 |  |
| 40. Frequency of health care use during past year | None =0, 1-5 =0,5, More than 5 =1 |  |
| 41. Number of prescribed medications Physical Performance and Anthropometry | None=0,1-4 =0.5, 5 and more =1 |  |
| 42. Body mass index | <18.5, ≥30=1   25-<30=0.5 26-18.5-25 =0 |  |
| 43. Handgrip strength | MALE:  For BMI≤24, GS≤29  For BMI≤24.1-28, GS≤30  For BMI>28, GS≤32=1 | FEMALE:  For BMI≤23, GS≤17  For BMI≤23.1-26, GS≤17.3  For BMI26.1-29, GS≤18  For BMI>29, GS≤21=1 |
| Laboratory Values |  |  |
| 44. Glycohemoglobin (%) | 0%-5.7%=0, >5.7%=1 |  |
| 45. Red blood cell count (million cells/mL) | M:4.7-6.1=0, Other=1 | F:4.2-5.4=0, Other=1 |
| 46. Hemoglobin (g/dL) | M:13.5-18=0, Other=1 | F:12-16=0, Other=1 |
| 47. Red cell distribution width (%) | 11.6-14.6=0, Other=1 |  |
| 48. Lymphocyte percent (%) | 20-40=0，Other=1 |  |
| 49. Segmented neutrophils percent (%) | 40-80=0，Other=1 |  |

**Q3:** I suggest that the authors specify which sampling weight was used to ensure the representativeness of the data, given that the study is based on a complex sampling design. Including this information would enhance the clarity and validity of the statistical approach.

**Response:** We sincerely apologize for not incorporating weighting in our original statistical analysis, and we fully understand the importance of this information, especially given the complex sampling design of our study. Prior to conducting our research, we reviewed numerous studies, many of which also did not apply weighting [1-6]. However, in response to your feedback, we have now incorporated sampling weights into our analysis. Detailed information on the weighting method, including the specific weights applied, has been added to the sensitivity analysis section of the manuscript. Additionally, the results of the weighted analysis have been included in the supplementary materials, where the weighting process is further elaborated. The inclusion of sampling weights was carefully considered, and we have verified that it does not significantly alter the overall conclusions of the study. Nevertheless, it does provide a more robust and representative analysis of the data. We have revised this part, and highlighted in red in the manuscript, and the revised text is transcribed below:

**Statistical analysis**

‘…. The mediation package in R 4.3.3 was used to examine the mediating association of serum 25(OH)D3 levels between dietary inflammation and frailty. Numerous studies have utilized publicly available data from the NHANES to investigate risk factors for various diseases. In these studies, some researchers have employed weighted analysis methods, while others have used unweighted approaches. Although NHANES employs complex sampling techniques to enhance the representativeness and generalizability of the survey results, discrepancies in conclusions may arise between weighted and unweighted analyses. In the present study, we conducted a sensitivity analysis using weighted logistic regression to reaffirm our findings. The weighting variable wtdrd2 were selected, and the combined weight was calculated as 1/2*wtdrd2. All sensitivity analyses were conducted using these weighted approaches.”

**Sensitivity analysis**

Similarly, the sensitivity analysis using weighted logistic regression indicated that when DII was treated as a continuous variable, a one-unit increase in DII was associated with a 9% increase in the risk of frailty in Model 3 (OR = 1.09, 95% CI: 1.01–1.18, P = 0.019). When DII was dichotomized (pro-inflammatory vs. anti-inflammatory), individuals with a pro-inflammatory diet had an 18% higher risk of frailty compared to those with an anti-inflammatory diet in Model 3 (OR = 1.18, 95% CI: 0.89–1.57, P = 0.216), although this association was no longer statistically significant. When DII was categorized into quartiles, individuals in the highest quartile (Q4) had a 62% higher risk of frailty compared to those in the lowest quartile (Q1) in Model 3 (OR = 1.62, 95% CI: 1.16–2.26, P = 0.006). (Table 6). The weighted mediation effect accounted for 6.8% of the total variance (0.0129/0.1886). (Table 7)

[1] Liu H, Tan X, Liu Z, Ma X, Zheng Y, Zhu B, Zheng G, Hu Y, Fang L, Hong G. Association Between Diet-Related Inflammation and COPD: Findings From NHANES III. Front Nutr. 2021 Oct 18;8:732099. doi: 10.3389/fnut.2021.732099. PMID: 34733875; PMCID: PMC8558221.
[2] Liu Z, Liu H, Deng Q, Sun C, He W, Zheng W, Tang R, Li W, Xie Q. Association Between Dietary Inflammatory Index and Heart Failure: Results From NHANES (1999-2018). Front Cardiovasc Med. 2021 Jul 6;8:702489. doi: 10.3389/fcvm.2021.702489. PMID: 34307508; PMCID: PMC8292138.
[3] Wu L, Shi Y, Kong C, Zhang J, Chen S. Dietary Inflammatory Index and Its Association with the Prevalence of Coronary Heart Disease among 45,306 US Adults. Nutrients. 2022 Oct 28;14(21):4553. doi: 10.3390/nu14214553. PMID: 36364813; PMCID: PMC9656485.
[4] Sun L, Huo X, Jia S, Chen X. The Association between Circadian Syndrome and Frailty in US adults: a cross-sectional study of NHANES Data from 2007 to 2018. Aging Clin Exp Res. 2024 May 7;36(1):105. doi: 10.1007/s40520-024-02745-3. PMID: 38713270; PMCID: PMC11076391.
[5] Huang Z. Association Between Blood Lead Level With High Blood Pressure in US (NHANES 1999-2018). Front Public Health. 2022 Apr 25;10:836357. doi: 10.3389/fpubh.2022.836357. PMID: 35548077; PMCID: PMC9081331.
[6] Azarmanesh D, Bertone-Johnson ER, Pearlman J, Liu Z, Carbone ET. Association of the Dietary Inflammatory Index with Depressive Symptoms among Pre- and Post-Menopausal Women: Findings from the National Health and Nutrition Examination Survey (NHANES) 2005-2010. Nutrients. 2022 May 9;14(9):1980. doi: 10.3390/nu14091980. PMID: 35565951; PMCID: PMC9105364.

Table 6 Weighted Logistic regression analysis of DII, 25-(OH)D3 and frailty.

| **Subgroup** | **Model 1** | | |  | **Model 2** | | |  | **Model 3** | | |
| --- | --- | --- | --- | --- | --- | --- | --- | --- | --- | --- | --- |
|  | **OR** | **95%CI** | ***P*** |  | **OR** | **95%CI** | ***P*** |  | **OR** | **95%CI** | ***P*** |
| **DII** | 1.17 | (1.11~1.24) | <0.001 |  | 1.11 | (1.04~1.18) | 0.001 |  | 1.09 | (1.01~1.18) | 0.019 |
| **DII** |  |  |  |  |  |  |  |  |  |  |  |
| <0 | Ref | 1 |  |  | Ref | 1 |  |  | Ref | 1 |  |
| >=0 | 1.65 | (1.33~2.06) | 0.001 |  | 1.35 | (1.03~1.75) | 0.027 |  | 1.18 | (0.89~1.57) | 0.216 |
| **DII Quantile** |  |  |  |  |  |  |  |  |  |  |  |
| Q1 | Ref. | 1 |  |  | Ref. | 1 |  |  | Ref. | 1 |  |
| Q2 | 1.44 | (1.05~1.96) | 0.023 |  | 1.16 | (0.81~1.67) | 0.374 |  | 1.13 | (0.78~1.64) | 0.473 |
| Q3 | 1.59 | (1.08~2.33) | 0.019 |  | 1.40 | (0.94~2.07) | 0.090 |  | 1.39 | (0.94~2.06) | 0.088 |
| Q4 | 2.19 | (1.66~2.89) | <0.001 |  | 1.68 | (1.22~2.32) | 0.003 |  | 1.62 | (1.16~2.26) | 0.006 |
| **25-(OH)D3** | 0.99 | (0.99~1.00) | 0.145 |  | 0.99 | (0.99~1.00) | 0.408 |  | 0.99 | (0.99~1.00) | 0.932 |
| **DII×25-(OH)D3** | - | - | <0.001 |  | _ | _ | 0.027 |  | 1.00 | (0.99~1.00) | 0.182 |

Model 1: unadjusted.

Model 2: adjusted for age, sex, education, PIR and BMI.

Model 3: Model 2+Physical activity, DBP, Total cholesterol, HDL and Energy.

S2 Table The relationship between DII and frailty was observed by subgroup analysis after weighting.

| **Subgroup** | **Model 1** | | |  | **Model 2** | | |  | **Model 3** | | |
| --- | --- | --- | --- | --- | --- | --- | --- | --- | --- | --- | --- |
|  | **OR** | **95%CI** | ***P*** |  | **OR** | **95%CI** | ***P*** |  | **OR** | **95%CI** | ***P*** |
| **Sex** |  |  |  |  |  |  |  |  |  |  |  |
| Male | 1.21 | (1.10~1.32) | <0.001 |  | 1.18 | (1.07~1.31) | 0.001 |  | 1.16 | (1.04~1.29) | 0.007 |
| Female | 1.12 | (1.02~1.22) | 0.012 |  | 1.05 | (0.96~1.16) | 0.232 |  | 1.04 | (0.93~1.18) | 0.404 |
| **Age** |  |  |  |  |  |  |  |  |  |  |  |
| [60~70) | 1.23 | (1.12~1.35) | <0.001 |  | 1.17 | (1.04~1.32) | 0.008 |  | 1.15 | (1.01~1.31) | 0.029 |
| [70~80] | 1.11 | (1.03~1.93) | 0.005 |  | 1.07 | (0.99~1.16) | 0.076 |  | 1.08 | (0.97~1.19) | 0.125 |

Model 1: unadjusted.

Model 2: adjusted for age, sex, education, PIR and BMI.

Model 3: Model 2+Physical activity, DBP, Total cholesterol, HDL and Energy.

|  | **β** | **SE** | ***p*** | **Bias-corrected 95%CI** | |
| --- | --- | --- | --- | --- | --- |
|  |  |  |  | **Lower** | **Upper** |
| Total effect | 0.1886 | 0.0228 | 0.0001 | 0.1349 | 0.23 |
| Direct Effects | 0.1757 | 0.0225 | 0.0001 | 0.1223 | 0.22 |
| Indirect Effects | 0.0129 | 0.0028 | 0.0001 | 0.0092 | 0.02 |

Table 7 Mediating relationship between DII and frailty in older adults after weighted 25-(OH)D3.

After controlling for age, sex, ethnicity, PIR, education, smoking, alcohol, sitting time, HDL, vitaminB12

**Q4:** In the Statistical Analysis section, I suggest including additional covariates that are relevant confounders in the relationship between vitamin D, frailty, and dietary inflammation. Specifically, I recommend incorporating an adiposity parameter, such as weight or preferably BMI, as well as a dietary intake parameter, such as total caloric intake. These variables would strengthen the analysis by accounting for important factors that may influence the observed associations.

**Response:** Thank you very much for your professional advice. In the current analysis, body mass index (BMI) and total caloric intake (Energy) have been included as covariates to more comprehensively account for potential confounding effects of BMI and dietary energy intake on the associations between vitamin D, frailty, and dietary inflammation. The results have been updated accordingly to reflect these adjustments. Detailed modifications to the methodology and revised findings are presented in the Results section. We have revised this part, highlighted in red in the manuscript, and the revised text is transcribed below:

**Relationship between dietary inflammation, Serum vitamin D and frailty**

Table 3 shows the results of the logistic regression of dietary inflammation index and frailty by three models. After adjusting for potential confounders (in Model 3), the results showed a significant association between DII and frailty, when DII was a continuous variable (OR=1.08, 95%CI [1.01~1.16], *p*=0.044). When DII was categorized into anti-inflammatory and pro-inflammatory diets, with the anti-inflammatory diet serving as the reference group, no significant association was observed between the pro-inflammatory dietary pattern and frailty (OR = 1.13, 95% CI: 0.80~1.62, *p*= 0.471). When DII was modeled as quantiles, it was found that after adjusting for confounding factors (in Model 3), compared to quartile Q1, the odds ratios (OR) for Q2, Q3, and Q4 were all greater than 1, but showed no statistical significance (p > 0.05). These results suggest that, after adjusting for confounding factors, there may be a positive association between DII and frailty. There was no statistical significance in the interaction between dietary inflammation and 25-(OH)D3 (*p*>0.05), Therefore, we suggest that 25-(OH)D3 may play a mediating role.

Table 3 Logistic regression analysis of DII, 25-(OH)D3 and frailty.

| **Subgroup** | **Model 1** | | |  | **Model 2** | | |  | **Model 3** | | |
| --- | --- | --- | --- | --- | --- | --- | --- | --- | --- | --- | --- |
|  | **OR** | **95%CI** | ***P*** |  | **OR** | **95%CI** | ***P*** |  | **OR** | **95%CI** | ***P*** |
| **DII** | 1.15 | (1.08~1.23) | <0.001 |  | 1.09 | (1.01~1.17) | 0.018 |  | 1.08 | (1.01~1.16) | 0.044 |
| **DII** |  |  |  |  |  |  |  |  |  |  |  |
| <0 | Ref | 1 |  |  | Ref | 1 |  |  | Ref | 1 |  |
| >=0 | 1.57 | (1.16~2.13) | 0.004 |  | 1.25 | (0.90~1.74) | 0.175 |  | 1.13 | (0.80~1.62) | <0.471 |
| **DII Quantile** |  |  |  |  |  |  |  |  |  |  |  |
| Q1 | Ref. | 1 |  |  | Ref. | 1 |  |  | Ref. | 1 |  |
| Q2 | 1.38 | (0.96~1.99) | 0.080 |  | 1.12 | ( 0.76~1.65) | 0.539 |  | 1.12 | (0.72~1.60) | 0.548 |
| Q3 | 1.57 | (1.09~2.24) | 0.014 |  | 1.32 | (0.90~1.95) | 0.150 |  | 1.36 | (0.83~1.90) | 0.117 |
| Q4 | 2.04 | (1.43~2.91) | <0.001 |  | 1.50 | (1.02~2.20) | 0.036 |  | 1.49 | (1.01~2.19) | 0.041 |
| **25-(OH)D3** | 1.00 | (0.99~1.00) | <0.132 |  | 0.99 | (0.99~1.00) | 0.744 |  | 1.00 | (0.99~1.00) | 0.932 |
| **DII×25-(OH)D3** | 1.00 | (1.00~1.00) | 0.004 |  | 0.99 | (0.99~1.00) | 0.129 |  | 0.99 | (0.99~1.00) | 0.410 |

Model 1: unadjusted.

Model 2: adjusted for age, sex, education, PIR and BMI.

Model 3: Model 2+Physical activity, DBP, Total cholesterol, HDL and Energy.

**Q5:** Table 1 (Participant Characteristics) is missing important parameters that would allow for a better understanding of the sample. I suggest including weight, height, BMI, and dietary intake variables, such as total calorie intake, protein, carbohydrates, total fats, and fat subtypes. These additions would provide a more detailed characterization of the study population and improve the overall interpretation of the findings.

**Response:** Thank you very much for your professional advice. In the Table 1 (Participant Characteristics), we have added relevant key parameters including weight (kg), height (cm), BMI, and dietary intake variables comprising total caloric intake (kcal), protein (gm), carbohydrates (gm), total fat (gm), and fat subtypes (gm). Additionally, we improved the overall description of the study findings. Detailed parameters are provided in Table 1 (Participant Characteristics) and the "Demographic Characteristics of Participants" section. These modifications have been highlighted in red in the manuscript, and the revised text is transcribed as follows:

**Demographic characteristics of participants**

“A total of 1172 participants were involved in the final study, of which 793 were non-frailty and 379 were frailty; males accounted for 46.59% of the sample, while females accounted for 53.41%. As shown in Table 1. Among all participants, the number of Pro-inflammatory diet (DII≥0) accounted for 76.28% of the total sample, and 309 people in the sample with high dietary inflammation index were frailty. Significant differences were observed between the frailty and non-frailty groups across demographic characteristics (age, sex, ethnicity, marriage, poverty income ratio [PIR]), clinical parameters (physical activity, sedentary time, total cholesterol, diastolic blood pressure [DBP], high-density lipoprotein [HDL], height, weight, body mass index [BMI], hypertension, diabetes), and nutritional profiles (energy, protein, carbohydrates, and Dietary Inflammatory Index [DII]) (all p < 0.05).”

Table 1	Demographic characteristics of participants (n=1172).

| **Subgroups** | **Total sample** | **Frailty** | | | ***p*** |
| --- | --- | --- | --- | --- | --- |
|  |  | Frailty | Non-frailty | |  |
| **Total sample (n (%))** | 1172 | 379（32.33） | | 793（67.66） |  |
| **DII (Mean ± SD)** | 1.46±1.92 | 1.80±1.81 | | 1.29±1.95 | <0.001 |
| **DII**  Anti-inflammatory  Pro-inflammatory | 278(23.72)  894(76.28) | 70(18.47)  309(81.53) | | 208(26.23)  585(73.77) | 0.003 |
| **Age (median (IQR))** | 68.00[64.00,74.00] | 70.00 [64.00, 77.00] | | 68.00 [63.00, 73.00] | <0.001 |
| **Sex (n (%))** |  |  | |  | <0.003 |
| Male | 546 (46.59) | 153(40.37) | | 393(49.56) |  |
| Female | 626 (53.41) | 226(59.63) | | 400(50.44) |  |
| **Ethnicity (n (%))** |  |  | |  | <0.021 |
| Mexican American | 72（6.14） | 20（5.28） | | 52（6.56） |  |
| Other Hispanic | 102（8.70） | 31（8.18） | | 71（8.95） |  |
| Non-Hispanic White  Non-Hispanic Black  Others | 651（55.55）  263（22.44）  84（7.17） | 216（56.99）  97（25.59）  15（3.96） | | 435（54.85）  166（20.93）  69（8.70） |  |
| **Education (n (%))** |  |  | |  | <0.073 |
| Less than 9th grade | 77（6.57） | 31（8.18） | | 46（5.80） |  |
| 9-11th grade | 145（12.37） | 51（13.46） | | 94（11.85） |  |
| High School graduate/GED or equivalent | 267（22.78） | 97(25.59) | | 170(21.44) |  |
| Associate or associate degree | 355（30.29%） | 110（29.02%） | | 245（30.90%） |  |
| College graduate or higher | 328（27.99%） | 90（23.75%） | | 238（30.01%） |  |
| **Marriage (n (%))** |  |  | |  | 0.002 |
| Be married | 708 (60.41) | 212（55.94） | | 496（62.55） |  |
| Unmarried | 52 (4.44) | 14（3.69） | | 38（4.79） |  |
| Divorce | 168（14.33） | 61（16.09） | | 107（13.49） |  |
| Others | 244（20.81） | 92（24.27） | | 152（19.16） |  |
| **Height (Mean ± SD (cm))** | 166 (158, 173) | 164±10 | | 167±10 | <0.001 |
| **Weight (Mean ± SD (kg))** | 78 (67, 91) | 83±21 | | 78±17 | <0.001 |
| **BMI(Mean ± SD)** | 27.9 (24.8, 32.0) | 30.8±7.1 | | 28.0±5.0 | <0.001 |
| **PIR (n (%))** |  |  | |  | <0.001 |
| <1.3 | 235（20.05） | 92（24.27） | | 143（18.03） |  |
| 1.3-3.49 | 474（40.44） | 177（46.70） | | 297（37.45） |  |
| >=3.5 | 463（27.99） | 110（29.02） | | 353（44.51） |  |
| **Smoking (n (%))** |  |  | |  | <0.113 |
| Yes | 573(48,89) | 198 (52.24) | | 375（47.29） |  |
| No | 599(51.11) | 181（47.76） | | 418（52.71） |  |
| **Alcohol consumption (n (%))** |  |  | |  | <0.180 |
| Yes | 822 (70.14) | 256(67.55) | | 566 (71.37) |  |
| No | 350 (29.86) | 123(32.45) | | 227 (28.63) |  |
| **Physical activity (n (%))** |  |  | |  | <0.001 |
| Inactive | 553 (47.18) | 140(36.94) | | 413(52.08) |  |
| Active | 619 (52.82) | 239(63.06) | | 380(47.92) |  |
| **Hypertension** |  |  | |  | <0.001 |
| Yes | 778 (66%) | 290(76.52%) | | 488(61.54%) |  |
| No | 394 (34%) | 89(23.48%) | | 305(38.46%) |  |
| **Diabetes** |  |  | |  | <0.001 |
| Yes | 260 (22%) | 126(33.25%) | | 134(16.90%) |  |
| No | 912(77.81% ) | 253(66.75%) | | 659(83.10%) |  |
| **Total fat(Mean ± SD (gm))** | 67 (44, 93) | 70±38 | | 73±37 | 0.116 |
| **Total saturated fatty**  **Acids (Mean ± SD (gm))** | 20 (13, 29) | 22±13 | | 23±13 | 0.190 |
| **Total monounsaturated fatty acids (Mean ± SD (gm))** | 23 (15, 33) | 25±15 | | 26±14 | 0.153 |
| **Total polyunsaturated fatty acids (Mean ± SD (gm))** | 15 (10, 23) | 17±11 | | 18±11 | 0.211 |
| **Sedentary time (median (IQR))** | 360.00(240.00,480.00) | 420.00(300.00,540.00) | | 360.00(240.00,480.00) | <0.001 |
| **SBP (median (IQR))** | 130.67(120.00,141.33) | 130.67(118.00,140.33) | | 130.67(120.67,142.00) | <0.458 |
| **DBP (median (IQR))** | 68.67 (61.33, 75.33) | 66.67 (58.00, 74.00) | | 70.00 (62.67, 76.00) | <0.001 |
| **Totalcholesterol(median(IQR))** | 4.84 (4.14, 5.64) | 4.68 (4.00, 5.46) | | 4.91 (4.19, 5.66) | 0.008 |
| **HDL (median (IQR))** | 1.37 (1.14, 1.66) | 1.34 (1.11, 1.63) | | 1.37 (1.16, 1.68) | 0.096 |
| **Vitamin B12 (median (IQR))** | 416.20(292.20,11.10) | 419.20(298.20,607.05) | | 415.50(289.65,612.9) | 0.918 |
| **Energy (Mean ± SD (kcal))** | 1742 (1299, 2253) | 1,740±694 | | 1,887±780 | 0.004 |
| **Protein (Mean ± SD (gm)** | 67 (49, 90) | 68±34 | | 74±33 | 0.001 |
| **Carbohydrate (Mean ± SD (gm)** | 207 (155, 273) | 208±80 | | 229±103 | 0.014 |
