# Supplementary material for: The serum vitamin D levels alleviate the influence of dietary inflammation on frailty: A cross-sectional analysis in the U.S. older adults
Source: PLoS One. 2025 Jun 30;20(6):e0327251. doi: 10.1371/journal.pone.0327251 (PMC12208477; doi:10.1371/journal.pone.0327251)
Supplement: Attachment — Submitted filename: Response_to_Reviewers_auresp_2.docx [file pone.0327251.s003.docx]

**Dear Editors and reviewers,**

**We sincerely appreciate your time and valuable comments, which have helped improve our manuscript. The submission ID is PONE-D-24-52566R2.**

**We hereby submit our responses to the reviewers' comments. We hope the revised manuscript now meets the publication standards of PLOS ONE.**

**Q1:** "Ratio of family income to poverty (RIP)" should be changed to "Ratio of family income to poverty (PIR)". In some places, still "RIP" is used for ratio of family income to poverty. Check throughout the manuscript.

**Response:** We sincerely appreciate the reviewer's careful reading. We have systematically replaced all instances of "Ratio of family income to poverty (RIP)" with the standard abbreviation "PIR" throughout the manuscript. We have made revisions to this part, highlighted in red in the manuscript, and the revised text is transcribed below:

Line 162: “….model 2 adjusting for sex, age, education, ~~RIP~~ PIR and ….”

Line 187: “….characteristics (age, sex, ethnicity, marriage, ~~RIP~~ PIR, clinical….”
